# Germinal Matter

**Published:** 1871-01

**Authors:** H. R. Noel


AMERICAN JOURNAL

OF

DENTAL SCIENCE.

Vol. IV. THIRD SERIES--JANUARY, 1871. No. 9.

### ARTICLE I.

### "*Germinal Matter.*"

**A Lecture delivered before the Class of the Baltimore College of Dental Surgery.**

**By Professor H. R. Noel, M. D.**

Gentlemen:—In the preceding lecture, the question of *Vitalitg and* "*The Vital Forces*" claimed our attention; to day I will but state the conclusions at which we arrived, and then pass to the discussion of the subject of "*Germinal Matter.*"

The first conclusion at which we arrived in the discussion of Vitality was this:

"*Vitality is inseparable from organization*"; and our next was that it was not an entity, not a thing distinct in itself, but the result of

"*Physical Forces passed through an Organic Germ*"; that there were only three manifestions of Vital Force, viz: (1) *Assimilative* (2) *Reproductive* (3) *Developmental*; and in these three expressions, the vegetable and animal kingdoms are one; mere vitality can give no other, and has no other expression of force; finally, that this element common to the two kingdoms, constructive in each and only constructive, had nothing whatever to do with

"*The animality or exhibition of Animal Forces*" found in the animal world, save 1st. *to construct*, 2nd. *to repair*

the waste produced by the working of those systems, which *it* had constructed and in virtue of whose workings the forces we term—*muscular*—*nerve*—and *brain force*, were obtained; this element or this active working agent is the only thing which can or which does exhibit the three expressions of Vital Force, therefore *Vital Force* is simply constructive, simply histogenetic, and Vitality belongs intrinsically to this Germinal Matter.

What then is *Germinal Matter?*

Anatomically it is the *Nucleus* of the older writers; it is the *cell* of Virchow and others; it is the *Nuclear matter* of Chambers of London; it is the *Protoplasm* of Huxley; it is the *Constructive Basis Element* of *Organic Life*; and again anatomically it is never larger than 1000th. of an inch, often much smaller, spherical—transparent or semi-transparent; minute speck,—a ball of jelly,—often faintly granular; watched under the microscope it seems, if fresh and warm, in constant motion; increases in size,—buds, divides; observing still more closely its action, we trace little currents of nutrient fluid to and from it, and find that one single mass may give origin to many others, all exactly alike, or nearly so, and all possessing the general outline, size, form and characteristics of the parent mass, and inheriting its vitality.

Where do we find these masses of Germinal matter?

The answer is plain, they are found wherever organic structure is to be built and wherever organic structure is to be repaired. As regards the human body, they are found in every tissue, every organ, every structure, and in each place and in every place they have but one office, one single function, that is, *Construction—Histogenesis.*

Primarily they produce the ultimate anatomical elements of all tissues, organs, and systems of organs; secondarily they repair all waste from either functional activity of tissue or organ, or waste from mere natural shedding or moulting; such as replacing our lost hairs, healing wounds, restoring our shaved beards, replacing nails torn off or crushed and lost by inflammatory action; so you will perceive they are never idle, eminently useful, always working either to restore a lost fragment or to construct a new element; in a word the very integrity of each tissue, each organ, each system, and the integrity of the organism as a whole, depends upon the sleepless vigilance of these microscopic masses, these balls of gelatinous material,—this Germinal Matter; or Constructive Basis Element of Organic Life.

In the blood they are called *white blooded globules;* in areolar or connective tissue they are called *connective tissue corpuscles;* upon the derm of skin and mucous membrane they are called *mucous globules;* in bone they are called *bone corpuscle* and *lacunæ corpuscles &c.;* but wherever found they are anatomically the same working factors, and possess no physical characteristics by which we can tell the tissue they are to produce; the Germinal Matter of bone, connective tissue—muscle—skin and mucous membrane &c., can not be distinguished, and therefore have no true anatomical differences, the masses in each tissue being apparently identical.

How then are special tissues produced?

This is a fair question, and a very natural one, and I am truly sorry I cannot answer it as fully as would be desirable; frankly, I cannot tell why masses apparently of identical composition, structure and physical characteristics should possess physiologically such diverse developmental powers. I will give you the facts, and the history of their developments, but I cannot explain *why* they possess these special manifestations of vital force; as a fact the tissues are produced "*in virtue of a special power inherent in and resident in the Germinal Matter of the special tissues*," this is all we know. This is an absolute,—an ultimate truth, susceptible of most practical demonstration as I will readily show you. If a piece of the periosteum of bone, be removed and implanted while still warm and fresh upon the skin, it will produce in its new home the same tissue it produced in its old home; in other words it will produce bone. The piece removed contains bone corpuscles, and these bone corpuscles have the special power of producing bone and not skin, hence when transplanted they retain that power and will actually live, grow, and develope but they *develope as bone.* The mucous globules of the skin possess the same powers, and may be transplanted successfully and be of great practical value, as the following statements will show; Prof. J. J. Chisolm, eminently the practical surgeon, and Dean of the Medical Faculty of the University of Maryland, had under his charge at their college hospital, some cases of intractable old ulcers; he exhausted his patience and skill upon these proverbially obstinate lesions, and finally considering them fit subjects for experimentation, he commenced a process of "skin budding," *i. e.* took snips of healthy skin and put them in little cuts and niches made in these old ulcers; now these snips of skin were fresh and warm—contained healthy mucous globules or masses of Germinal Matter, they immediately, pardon the phrase, *took root*, so to speak, and grew off, giving him numerous islands of healthy skin in this denuded surface; the islands increased in size, united their edges, and he absolutely *skinned over* these ulcers by this process of skin grafting; or skin budding; he cured them, healed them up by virtue of the special power inherent in the Germinal Matter of special tissues. This may appear strange, but you will be more surprised when I tell you that many of your fathers have performed the same operation; you are aware that among farmers and horticulturists there is a process known as *budding* trees &c., now this is nothing more than M. Ollier did in his skin grafting or budding, or than Prof. Chisolm in his *skin budding* of ulcers, has done in the animal kingdom—the two processes are physiologically identical and depend upon the truth above stated.

Bone diseased by cancer will often give rise to cancer in the lungs, liver, &c., but always to "bone cancer," for the vessels ulcerating through, take up the bone corpuscles tainted with cancer, and, wherever in the circulation these may be arrested, they are liable to give rise to both bone and cancer; for they preserve intact, though transported to distant organs, the power to produce bone and the power to produce cancer.

In that terrible disease syphilis, it is probable that the phenomena of its secondary or constitutional effects, can be explained upon this very principle; each diseased gland, or ulcerating surface might act as a depot of infection and further contamination. From these facts you can at once perceive, how important a truth this is and how essential that we should know at least the condition and laws of its manifestations though we are utterly unable to explain the *whys* of such phenomena; we cannot determine the *final causes*, but we may attain a knowledge of the physical conditions, and physiological and pathological laws which govern the phenomena.

Special tissues then result from special powers resident in the Germinal Matter of these tissues, and each tissue is the result of the expression of the 3d., or developmental power of the Germinal Matter; the masses from bones, muscles, tendons, mucous membrane &c., have the 3rd. power, the product of its exhibition varies with the tissue examined, and there are no anatomical characters by which we can by an antecedent examination determine what this product will be.

THE SIZE OF GERMINAL MATTER.

I stated that this organic basis element never attained a size larger than 1000th. of an inch, and might be almost immeasurably small; now a doubt might arise as to the ability, of so small a working agent, to produce the different tissues, organs &c.; this objection is more apparent than real, for a microscopic analysis of the ultimate anatomical tissue elements, reveals the fact, of a striking relationship as regards size, which the following statement will verify:

Ultimate Fibrillæ of muscle 5000th. to 10000th. of an inch. Ultimate Nerve Fibrillæ 1000th. to 10000th. of an inch. Blood Globules, 2000th. to 3400th.; Ossicle of bone 500th. to 2500th. Enamel Rods or Columns 5000th. of an inch; Dentinal Fibrillæ 10000th. to 20000th.; and other elements in the same ratio; now these elements make up the animal fabric, for it is by a multiplication of similar elements and not by the introduction of new ones, that the body is constructed; for example ultimate, fibrillæ of muscle 10000th. of an inch in diameter, packed in a sheath, called *Sarcolemma*, will give an ultimate fibre 350th. to 1000th. of an inch in diameter; these fibres connected by a fine connective tissue give fasciculi; these fasciculi in bundles give complete muscles. The history of the physical structure of muscle is the history of all complex tissues, they are all built of microscopic elements, blended, repeated, interwoven, until we get structures as different from the original elements, as the richest brocade silks are from the raw material, just removed from the cocoon of the bombyx mori—or moth of the silk worm.

Therefore, since it is by a repetition of smaller elements, that we get the larger anatomical forms, the size of germinal matter so far from being against the truths I have stated, is in fact a most striking agument in their favor; this microcroscopic factor, in virtue of the three powers characteristic of vegetative life **, is fully competent to construct the elements of which the human organism is composed, and being competent for its primary construction they are equally competent for repair of injury, waste, or normal moulting of substance by molecular disintegration.

In general terms then, it may be said that all naked eye anatomical elements are composed of microscopical elements, and these latter elements have been derived directly from the Vital Force of Germinal Matter. ***Assimilation—Reproduction—Developement.*

THE CARMINE TEST OF DR. LIONEL S. BEALE.

In a former lecture, I told you that the histogenetic agent and the animal working elements of muscle were by no means identical, and possessed very different powers; the first is of course the Germinal Matter, and the latter is the muscular fibre composed of ultimate fibrillæ; the one possesses the three vegetative powers, *assimilative—reproductive* and *developmental ;* the other, i. e. the fibre has the one single power of "*contractility*" and gives us by this power "Muscular Force," but it has nothing to do with the vegetative Forces; it cannot repair its own waste, and so far as *nutrition* is concerned it is perfectly passive, but so far as *motion* is concerned it is eminently active; the germinal matter and the formed tissues have in this instance totally different functions, and the one never could assume the other's position. When germinal matter exhibits its developmental power, it ceases to be germinal matter, and in that very act becomes changed into a formed tissue, which may have other and perhaps higher functions, but which can never again be germinal matter, therefore never again exhibit the Vegetative Force. This being true of germinal matter and formed tissue in muscle, it will be found true of them in all tissues, or structures of the body.

Can these two things be accurately distinguished? Dr. Beale answes this in the affirmative, and gives numerous diagrams representing a great variety of tissues in proof of his position. He uses a preparation of carmine which stains red the *Germinal Matter*, but does not affect the formed tissue, or effects it so very slightly that it can be readily removed. He uses two terms to distinguish the histogenetic factor and the material this factor elaborates, he calls the one, as I have already told you, *Germinal Matter*, he calls the other Formed Material; so all tissue is Formed Material with Dr. Beale; he says "*Germinal Matter removed from any organism which was recently alive, is tinged red with carmine, and preserves the color permanently when preserved in glycerine. Formed Material is not colored, but if it be stained, owing to the solution* (*carmine*) *being too strong the stain is removed by soaking in glycerine*;" from this you will percieve that he makes an accurate yet simple distinction between them. Dr. Beale having obtained his means of distinguishing the two, proceeded to examine under the microscope the different tissues of the body and traced out by close observation from comparative anatomy and rigid experimentation, the evolutions of the varions ultimate anatomical elements, and demonstrated their relations to the germinal matter in so clear and so forcible a manner, that we are now in the possession of information as to the histogenesis of the human body, which will radically change both modern physiology and pathology.

He conclusively proves the nutritive or histogenetic activity of germinal matter, and the complete passivity of Formed Material, so far as nutritive action is concerned; he proves that a muscular fibre once formed, remains, so far as vegetative force is involved, perfectly passive—it may contract—it may slowly disintegrate by molecular change, but it never gives one single proof, or one particle of evidence of being *alive*, so far as the vegetative forces may be considered as an evidence of life; the fibre never assimilates, never propagates, never develops itself, but remains quiescent as regards Vital Force, until it is worn away by use, or destroyed by gradual moulting. I candidly confess, that there is just at this point, some rather nebulous ground, and I will state it freely, it is this: *as given by Beale*, *Huxley*, *Chambers*, *&c.*, *Germinal matter alone possesses Vitality*, while the Formed Material, though itself the result of the expression of the third power, or highest possible expression of Vital Force, is not really *alive*, is not vegetatively alive, but remains perfectly passive in respect to this force. This Formed Material may have high powers, as muscular, nerve and brain force for example; or its function may be a purely passive or even a mechanical one, as the hair, nails, bone, cartilage, enamel, dentine, &c.; now these structures, tissues, etc., being nothing more than the formed material of the germinal matter, and having no claim to any vital quality or property (or expression of vitality), yet remain often months after their formation before they are moulted or used up in the functional activity of the systems of animal life, and during this interval are component parts of the organism. How shall we designate them, if not alive are they dead ? If dead why do they not at once undergo putrefaction ? Why if indeed not alive, do they not give us the evidences of death, i. e. rapid chemical change ? The formed tissues certainly never return again to the state of *basis* or constructive element, and therefore can never give any evidence of Vegetative Force, thus they are not alive, in the sense in which the *word* is used in reference to the vegetative kingdom, yet they are as regards the animal, the very machinery by which it is enabled to give evidence of its animality, "hence are as necessary to an expression of animality" as germinal matter is to the expression of vitality ; the question therefore, as it now stands, can only be properly understood and scientifically discussed by making a sharp distinction between the *constructive factor* and the *machinery* which it constructs ; to the working constructive agent we assign vitality, to the constructed machinery we assign *animalty* Now the animal machinery once constructed remains in working order about four or six months and then if not repaired or replaced by new material it begins to undergo a degeneration; one of the first is a *fatty change.* It is probable that all nitrogenous tissues are renewed every four months ; four months is their healthy term, and after this they must either be renewed, or the machinery suffers from direct degeneration of the material of which it is composed. Machines of wood, iron, steel, silver, gold, &c., last often for many years, requiring repair only at long intervals, but yet all undergo more or less of constant wear or loss by use; and in the working of the animal machinery there is also wear or loss by use ; this wear is incessant during the activity of the animal, it is molecular or interstitial, or as I might better express it, *atom by atom* ; this atom by atom loss, whether from functional activity or normal moulting, has to be met by a coincident or nearly coincident atom by atom repair, else the integrity of the machinery suffers ; for this repair rest and food are necessary, *i. e.* time and material.

Practically, you have often acted upon this very truth; you would not drive a horse for two consecutive days and nights without rest or food; you would not drive him eighteen consecutive hours without rest or food, unless the case were one of extreme emergency, and then you would feel that you were doing the animal an unavoidable wrong; you would be guilty of excessive waste of animal machinery without giving time or material for repair; you may use your watch for twelve months without repair, you cannot use any animal machinery one day without repair, except with detriment to the animal. The physical machine is repaired by the proper mechanician, but the animal machinery carries its own element of repair as well as of primary construction; the germinal matter, the nuclear matter, the proto-plasm, the basis element of organic or vegetative life.

Vitality and Animality are widely different, but Animality is directly dependant upon the vitality of the histogenetic element, which primarily constructs and afterwards maintains the integrity of the machiney in virtue of whose action, Animality exists.

